# The ability of the Lab4 probiotic consortium to impact upon the functionality of serum deprived human keratinocytes *in vitro*

**DOI:** 10.3389/frmbi.2024.1488650

**Published:** 2024-11-19

**Authors:** Sophie E. Thomas, Joshua Kerry-Smith, Susan F. Plummer, Jack P. Bate, Daniel A. John, Evie Lawrence, Lydia Powell, Jordanna Dally, Ryan Moseley, Daryn R. Michael

**Affiliations:** ^1^ Research and Development, Cultech Limited, Port Talbot, United Kingdom; ^2^ Department of Clinical and Biomedical Sciences, Faculty of Health and Life Sciences, University of Exeter, Exeter, United Kingdom; ^3^ Microbiology and Infectious Diseases, Institute of Life Sciences 1, Swansea, United Kingdom; ^4^ Disease Mechanisms Group, Oral and Biomedical Sciences, School of Dentistry, Cardiff University, Cardiff, United Kingdom

**Keywords:** keratinocyte, serum deprivation, probiotic, gut-skin axis, Lab4, conditioned media

## Abstract

**Introduction:**

Dysfunction of keratinocytes contributes to a weakened skin barrier and impaired wound healing capability. Evidence suggests that probiotic supplementation can lead to improved skin function in vitro and in vivo. The Lab4 probiotic consortium comprises of two strains of Lactobacillus species and two strains of Bifidobacterium species.

**Methods:**

Using serum deprived conditions to impair the functionality of immortalized human HaCaT keratinocytes, this study aimed to assess the impact of metabolites derived from the Lab4 probiotic consortium on keratinocyte function.

**Results:**

A significant improvement in HaCaT metabolic activity and lower apoptotic activity was observed in tandem with a reduction in Caspase-3 gene expression and a lower Bax/Bcl2 ratio following the addition of Lab4. The probiotic also supported barrier integrity which was better maintained with a significant increase in Filaggrin gene expression. In damaged keratinocytes, Lab4 enhanced rates of re-epithelialization, which were associated with significantly increased gene expression of MMP-1 and enhanced secretion of IL-6 and IL-8.

**Discussion:**

These results suggest that the Lab4 probiotic consortium may have the ability to benefit the functionality of skin.

## 1 Introduction

Skin provides a protective, frontline barrier against environmental agonists such as pathogens, allergens and chemicals. It also plays a role in facilitating permeability and moisture retention. The key structural component of skin that facilitates functionality is the outermost epidermal layer known as the stratum corneum, that is comprised of keratinocytes (Menon et al., 2012). Key functions of keratinocytes are to form a tight barrier and facilitate wound healing (Menon et al., 2012) and loss of function caused by numerous stress factors including aging, the environment and/or nutrient deficiency (poor diet) can lead to increased risk of infection and disease (Woo and Kim, 2024; Ahmed and Mikail, 2024). The HaCaT keratinocyte cell line (Boukamp et al., 1988) is extensively used to study epidermal homeostasis and pathophysiology *in vitro* (Blanchard et al., 2022).

It is becoming recognized that the gut microbiome - the trillions of micro-organisms residing in the intestinal lumen - contributes to skin health and function via the ‘gut-skin axis’ and alterations in the gut microbiome composition such as a lack of diversity and lower abundance of beneficial bacteria have been linked to a number of skin disorders including atopic dermatitis (Liu et al., 2023), psoriasis (Codoñer et al., 2018) and acne vulgaris (Yan et al., 2018). Oral supplementation with probiotic bacteria - defined as “live microorganisms which when administered in adequate amounts confer a health benefit on the host” (Hill et al., 2014) - has been associated with beneficial skin effects. Strains of *Lactobacillus* species have shown an ability to reduce symptoms of atopic dermatitis in humans (Rather et al., 2021; Yamamoto et al., 2016), while *Bifidobacteria* have shown the capability to improve skin barrier integrity in mice (Kim et al., 2022) and ameliorate age-related skin damage in humans (Huuskonen et al., 2022). The underlying mechanisms are not yet understood but it is thought that these effects may be mediated, at least in part, by the translocation of bacterial metabolites from the gut to the skin via the circulatory system (Wilson et al., 2020).

The Lab4 probiotic consortium, comprising *Lactobacilli* and *Bifidobacteria*, has been shown to support the viability and function of numerous peripheral cell types *in vitro* (Michael et al., 2019, Davies et al., 2018; O'Morain et al., 2023), including serum deprived neurons (Webberley et al., 2023), and holistic benefits have been observed in human intervention studies (Mullish et al., 2024, 2023, John et al., 2024; Paduchová et al., 2024; Hepburn et al., 2013; Williams et al., 2009). The ability of Lab4 to support skin health and functionality is yet to be explored.

In this *in vitro* study, serum deprivation was used to impair the metabolic activity, barrier function and re-epithelialization capability (wound healing) of HaCaT keratinocytes in order to assess the potential of metabolites generated by the Lab4 consortium to support skin functionality during challenged conditions.

## 2 Materials and methods

### 2.1 Materials and reagents

All tissue culture and molecular reagents were purchased from ThermoFisher Scientific (Paisley, UK), unless otherwise stated. All microbiological reagents were purchased from Oxoid (Basingstoke, UK), unless otherwise stated.

### 2.2 HaCaT cell culture

Human derived immortalized HaCaT keratinocyte cells (gifted by Professor Ryan Moseley’s group, Cardiff University, Wales) were maintained in Dulbecco’s Modified Eagle’s Medium (DMEM) containing 10% (v/v) fetal bovine serum (FBS) and 100 U/mL penicillin/streptomycin, in a humidified incubator (Heraeus, Hanau, Germany) at 37 ˚C and 5% CO₂. Cells of ~80% confluency were seeded into appropriate tissue culture plates at a density of 7.8 x 10^4^ cells/cm² and incubated for 24 h to reach 100% confluency.

### 2.3 Preparation of Lab4 conditioned media

The Lab4 probiotic consortium comprises: *Lactobacillus acidophilus* CUL21 (NCIMB 30156), *L. acidophilus* CUL60 (NCIMB 30157), *Bifidobacterium bifidum* CUL20 (NCIMB 30153) and *B. animalis* subsp*. lactis* CUL34 (NCIMB 30172). Each strain was grown anaerobically overnight (18 h) at 37 °C in MRS (de Mann Rogosa and Sharpe) broth for CUL21 and CUL60 and MRS supplemented with 0.25 g/L cysteine hydrochloride for CUL20 and CUL34. The viable numbers were determined according to the method of Tracey et al. (2023) and each culture was centrifuged (4000 x g for 10 min), washed in sterile phosphate buffered saline (PBS), re-centrifuged and re-suspended in PBS. The Lab4 probiotic consortium (1 x 10^8^Active Fluorescent Units (AFU)/ml) was constructed from these bacterial suspensions, mixed, centrifuged and the pellet was re-suspended in pre-reduced DMEM and incubated anaerobically (37 °C/18 h) to generate Lab4 conditioned medium (L4CM). There was no increase in bacterial numbers overnight. The L4CM was harvested, pH adjusted to 7.4, supplemented with 100 U/mL penicillin/streptomycin prior to filter sterilization (0.2 μm), aliquoted and stored at -70 °C. The L4CM was inoculated onto confluent HaCaT cells at 0.5 ml L4CM representing 5 x 10^7^ bacterial AFU per 1.9 cm^2^ of HaCaT cells (representing 1.85 x 10^5^ cells) providing an application rate of probiotic to keratinocyte of ~270:1. For optimization experiments, doses of L4CM were created by mixing with DMEM and are expressed as volume to volume (v/v) dilutions.

### 2.4 Assessment of HaCaT metabolic activity

Confluent monolayers of HaCaT cells (grown for 24 h post-seeding) were exposed to decreasing levels of serum between 10% (representing ‘healthy’ conditions) to 0% (serum free conditions) and after a 48 h incubation (at 37 °C and 5% CO₂), metabolic activity (considered an indicator of viability) was assessed using the 4,5-dimethylthiazol-2-yl)-2,5-diphenyltetrazolium bromide (MTT) assay (Michael et al., 2019). Metabolic activity was expressed as a percentage in relation to the 10% serum control that was designated as 100%.

### 2.5 Apoptosis/necrosis assay

HaCaT cells were seeded into black sided, clear bottomed 96-well microplates (Greiner, Kremsmünster, Austria) and incubated overnight to allow for adherence (at 37 ˚C and 5% CO₂). Levels of necrosis and apoptosis were measured in the HaCaT cells exposed to experimental conditions (delivered in phenol red-free media to eliminate interference) using the RealTime-Glo™ Annexin V Assay kit (Promega, Wisconsin, USA) in accordance with manufacturer’s instructions (Kupcho et al., 2019). Levels of necrosis are presented as relative fluorescence units (RFU) and levels of apoptosis are expressed as relative luminescence units (RLU).

### 2.6 Gene expression analysis

Total RNA was isolated from the HaCaT cells using TRIzol™, quantified using a Qubit 2.0 fluorometer and cDNA generated from 1 µg of total RNA using the High Capacity cDNA Reverse Transcription Kit, all carried out in accordance with manufacturer’s instructions. Realtime PCR was performed on 200 ng of cDNA according to Michael et al. (2016) using a QuantStudio™ 5 Real-Time PCR System. Oligonucleotide primer sequences are shown in **Supplementary Table S1**. Relative gene expression was calculated using 2^-(ΔCt1 – ΔCt2)^, where delta Ct represents the difference between the cycle threshold (Ct) for the target gene (Ct1) and the house keeping gene (β-Actin) (Ct2). Gene expression data were expressed as mean fold changes in comparison to the appropriate experimental controls that have been designated as 1.

### 2.7 Measurement of barrier integrity using trans epithelial electrical resistance

HaCaT cells were grown to confluency on 0.4 μm pore PET membrane trans-well inserts (Thincert™, Greiner, Kremsmünster, Austria) in 24 well plates. Trans epithelial electrical resistance (TEER) (ohms) was measured using an EVOM2 Epithelial Voltohmeter STX2 chopstick electrode set (World Precision Instruments, Hertfordshire, UK) in accordance with manufacturer’s instruction (Srinivasan et al., 2015). Prior to experimentation, and between technical replicates, the electrode was sterilized by immersion in ethanol and then in sterile DMEM. TEER was calculated as ohms*cm^2^ after correction for the background reading of a cell free trans-well insert and are presented as percentage change in comparison with the appropriate experimental control that has been designated as 100%.

### 2.8 Assessment of the impact of L4CM on scratch wounding

Confluent HaCaT monolayers in 24 well plates were scratched using a standard 200 µL pipette tip (method adapted from Vang Mouritzen and Jenssen (2018)), producing a single vertical lesion (approximately 550 μm in width) spanning the diameter of the well for migration assays (section 2.8.1) or 3 vertical and 3 horizontal lesions spanning the well for assessment of IL-6 and IL-8 protein levels (section 2.8.2) and gene expression (section 2.6). After scratching, cells were washed twice with PBS to remove debris before the addition of experimental conditions.

#### 2.8.1 Re-epithelialization assay

Scratches were imaged over 48 h (at 4 X magnification) using a DMi1 Inverted Light Microscope with camera (Leica, Wetzlar, Germany). From the images, the area of the scratch (μm^2^) was measured using the “Wound healing size tool” plug-in in ImageJ (Suarez-Arnedo et al., 2020). Rates of cell migration or ‘wound healing’ were calculated according to the equation (Jonkman et al., 2014):


$$Vmigration=\frac{\left[slope\right]}{2\;x\;l}$$

Here, *Vmigration* is average velocity of cell movement. The *slope* value is area of the wound (μm²) over time (h) and *l* refers to length of the region of interest (1671.82 μm for all images, as calculated by ImageJ). Values were calculated as μm/h and expressed as percentage change relative to the appropriate experimental control that has been designated as 100%.

#### 2.8.2 IL-6 and IL-8 enzyme linked immunosorbent assay

Cell supernatants were collected 4 and 48 h after scratching and centrifuged at 2000 x g for 10 minutes, to remove cell debris. Supernatants were analysed for IL-6 and IL-8 protein concentration using the Human IL-6 SimpleStep Enzyme Linked Immunosorbent Assay (ELISA^®^) Kit (ab178013) and the Human IL-8 SimpleStep ELISA^®^ Kit (ab214030) (both Abcam, Cambridge, UK) in accordance with manufacturer’s instructions and as outlined in Lin et al. (2024). The remaining cell monolayers were processed for gene expression analysis as per section 2.6.

### 2.9 Statistical analysis

Normality of data was assessed using the Shapiro-Wilk test and by visual inspection of Q-Q plots to confirm normal distribution. Differences between groups were analysed using an unpaired parametric Student’s t-test (for single comparisons), One-way analysis of variance (ANOVA) with Dunnett’s *post hoc* testing (for multiple comparisons) or Two-Way ANOVA, followed by Tukey’s *post hoc* testing (for repeated measure multiple comparisons). Differences were determined to be significant where *p≤*0.05. All statistical analysis was performed using GraphPad (Prism, Version 10, Boston, USA). All data are presented as the mean ± standard deviation of at least 3 independent experiments, unless otherwise stated.

## 3 Results

### 3.1 L4CM preserves metabolic activity in serum deprived HaCaT cells

**Figure 1:**
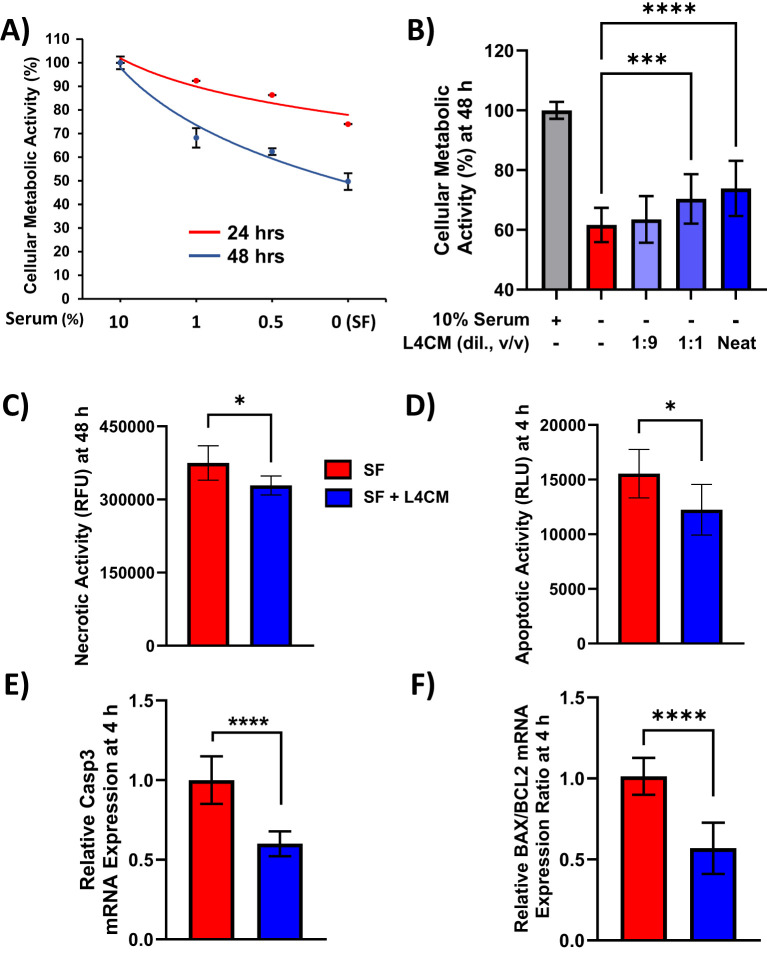
L4CM enhances metabolic activity and reduces apoptotic activity in serum free HaCaT cells. HaCaT cell metabolic activity with **(A)** decreasing concentrations of serum for up to 48 h. **(B)** HaCaT cells incubated with 10% (v/v) serum, without serum (serum free, SF) or SF with various dilutions (v/v) of L4CM for 48 h. Data are expressed as percentage in relation to 10% serum treated cells (set to 100%). **(C)** Necrotic activity in HaCaT cells after 48 h and **(D)** early apoptotic activity after 4 h of serum free HaCaT cells with or without neat L4CM supplementation. Data are expressed as the mean relative fluorescent units (RFU, for **C**) or mean relative luminescent units (RLU, for **D**). Relative gene expression levels of **(E)** Caspase-3 and **(F)** BAX/BCL2 in SF HaCaT cells supplemented with or without neat L4CM for 4 h. Data are expressed as fold change in relation to the SF control (set to 1). Values of *p* were determined using One-Way ANOVA followed by Dunnett’s *Post Hoc* analysis (for **B**) or an unpaired two-way Student’s t-test (for **C**–**F**) where *p<0.05, ***p<0.001 or ****p<0.0001. Bax, Bcl-2-associated X apoptosis regulator; Bcl-2, B-cell lymphoma 2 gene; Caspase-3, cysteine-aspartic protease 3.

The metabolic activity of HaCaT cells exposed to decreasing concentrations of serum was assessed and indicated ~50% loss of activity after 48 h of serum-free (SF) conditions in relation to cells incubated with 10% serum (**Figure 1A**). When SF cells were incubated with varying concentrations of Lab4 conditioned medium (L4CM) for 48 h there was a dose related increase in metabolic activity from 59% in the SF cells to 60% with the addition of a 1:9 dilution of L4CM, 67% (*p*=0.0007) with a 1:1 dilution of L4CM and 71% (*p*<0.0001) with neat (undiluted) L4CM (**Figure 1B**).

Metabolic activity is commonly used as an indicator of cellular viability and necrotic activity in cells exposed to neat L4CM for 48 h was 12% lower (*p*=0.0189, **Figure 1C**) compared to SF cells. For all subsequent experiments, L4CM was applied neat and is referred to as ‘L4CM’ hereafter. Necrosis is preceded by apoptosis and the highest levels of apoptosis were recorded in HaCaT cells after 4 h of serum deprivation (**Supplementary Figure S1**) when 21% less apoptotic activity was detected with L4CM compared to the SF control (*p*=0.0301, **Figure 1D**). The lower apoptotic activity observed at 4 h was associated with lower expression of the pro-apoptotic Caspase-3 gene (0.6-fold change, *p*<0.0001, **Figure 1E**) and a lower ratio of apoptotic markers Bax/Bcl-2 (0.57-fold change, *p*<0.0001, **Figure 1F**) when compared with SF cells.

### 3.2 L4CM preserves barrier integrity

**Figure 2:**
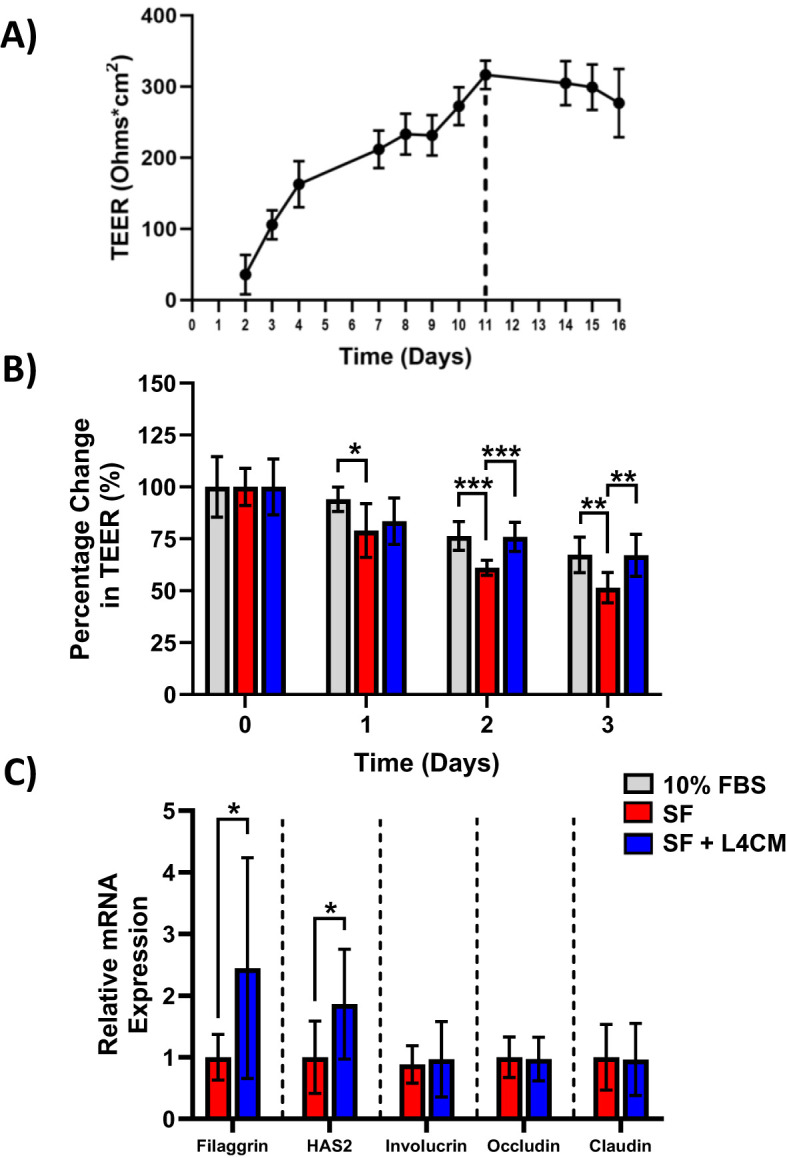
The impact of L4CM on barrier integrity in serum free HaCaT cells. **(A)** Temporal changes in TEER (ohms*cm^2^) in HaCaT cells maintained with daily feeding for 16 days. Data represents mean ± SD of 2 independent experiments. **(B)** Changes in TEER of 11-day old HaCaT cells after exposure to 10% serum or serum free (SF) conditions with or without L4CM for 72 h. Data is expressed as mean percentage change compared to 10% serum (set at 100%). **(C)** Relative gene expression levels of filaggrin, HAS2, involucrin, occludin, and claudin in SF HaCaT cells with or without exposure to L4CM for 48 h. Data are expressed as a fold change in relation to the SF control (set to 1). Values of *p* were determined using repeated measures two-way ANOVA with the Geisser-Greenhouse correction and Tukey’s *post-hoc* (for **B**) or unpaired parametric students t-test (for **C**) where *p<0.01, **p<0.01 or ***p<0.001. Filaggrin, filament aggregating protein; HAS2, hyaluronan synthase 2.

Maximum HaCaT TEER was reached after 11 days of incubation as shown in **Figure 2A**. Subsequently, HaCaT cells were incubated for 11 days and the effect of serum deprivation on the cells was subsequently assessed with and without the addition of L4CM over 3 days (**Figure 2B**).

The barrier integrity, as measured by the TEER of the SF HaCaT cells, decreased more over time in comparison with cells supplemented with 10% serum with a significant reduction in TEER of 16% observed after day 1 (*p*=0.0378), 20% after day 2 (*p*=0.0004) and 23.5% after day 3 (*p*=0.0036) in comparison. Levels of TEER for the 10% serum control and the L4CM supplemented cells remained similar throughout the 3 days.

The barrier integrity of HaCaT cells was found to be significantly better than that of SF challenged cells with the inclusion of L4CM over the 3 days. The SF cells showed greater deterioration of barrier integrity over time in comparison with cells supplemented with L4CM with a 5.7% decrease in TEER observed after day 1, 24.4% (*p*=0.0005) after day 2 and 30.2% (*p*=0.01) after day 3 in comparison.

Assessment of the barrier integrity related genes Filaggrin, HAS2, Involucrin, Occludin and Claudin indicated significantly higher expression levels of Filaggrin (2.44-fold change, *p*=0.0124, **Figure 2C**) and HAS2 (1.86-fold change, *p*=0.0131) in L4CM supplemented cells compared to SF cells after 48 h. Levels of Involucrin, Occludin and Claudin showed no significant differences.

### 3.3 L4CM improves re-epithelialization

**Figure 3:**
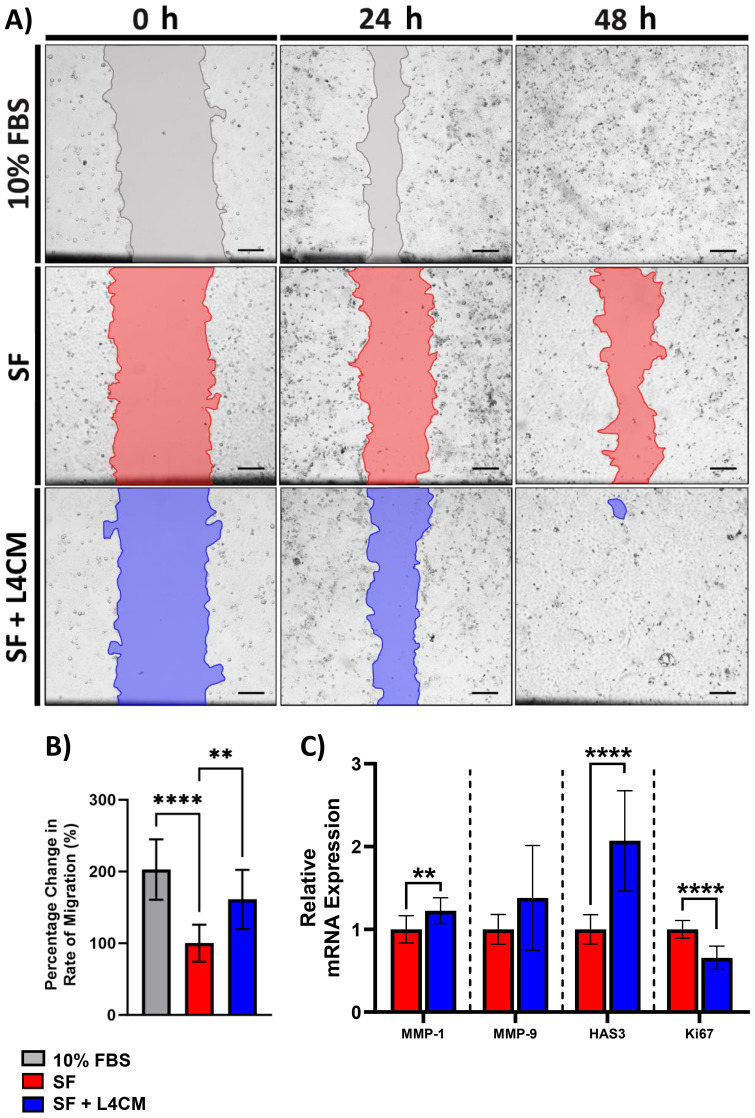
The impact of L4CM on HaCaT re-epithelialization under in serum free cells. **(A)** Representative images of scratched HaCaT monolayers incubated with 10% serum, or serum free (SF) conditions with or without L4CM supplementation for 48 h (4X magnification, scale bar = 150 µm). **(B)** Change in rate of HaCaT cell re-epithelialization in scratched HaCaT cells exposed to 10% serum or SF conditions with or without L4CM for 48 h. Data are expressed as a percentage change in relation to the SF control (set to 100%). **(C)** Relative gene expression of MMP-1, MMP-9, HAS3 and Ki-67 in scratched SF HaCaT cells with or without a 4 h incubation with L4CM. Data are expressed as fold change compared to SF cells (set to 1). Values of *p* were determined using an unpaired parametric Student’s t-test where **p<0.01 or ****p<0.0001. MMP-1, matrix metalloproteinase 1; MMP-9, matrix metalloproteinase-9; HAS3, Hyaluronan Synthase 3.

Confluent monolayers of HaCaT cells were damaged by “scratching” and the rate of cell migration to close the denuded area (to achieve re-epithelialization) was monitored over 48 h (**Figure 3A**). Compared to the 10% serum control, rate of migration in the SF cells was 49.5% lower (*p*<0.0001, **Figure 3B**). In L4CM supplemented cells, the rate of migration was 61% higher (*p*=0.0036, **Figure 3B**) than seen with the SF cells and was comparable to that measured in the 10% serum control (*p*=0.0646).

Gene expression analysis 4 h after scratching indicated significantly higher levels of MMP-1 (1.22-fold change, *p*=0.0089, **Figure 3C**) and HAS3 (2.1-fold change, *p*<0.0001) in the L4CM supplemented cells compared to the SF control, but no changes were observed in MMP-9 (*p*=0.0868). Expression of the proliferation marker Ki-67 was significantly lower (0.66-fold change, *p*<0.0001) in L4CM cells compared with the SF control.

### 3.4 L4CM impacts the inflammatory response of scratched HaCaT cells

**Figure 4:**
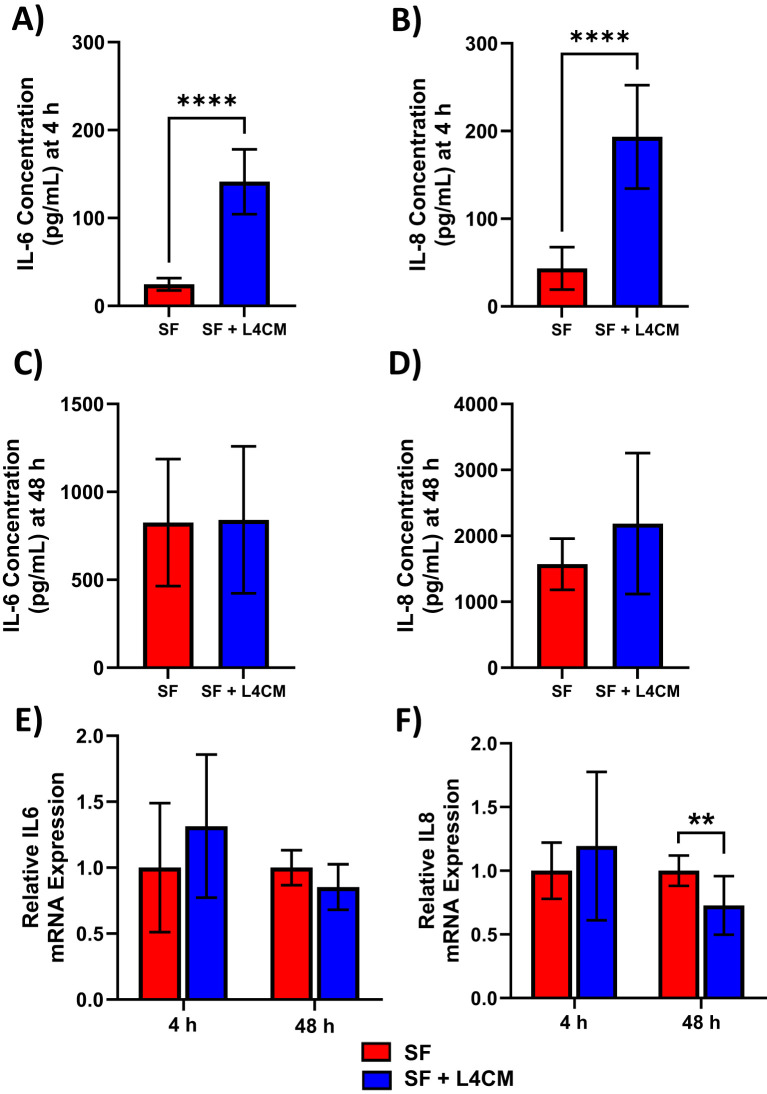
L4CM modulates IL-6 and IL-8 protein production in scratched, serum free HaCaT cells. Protein concentration of inflammatory cytokines IL-6 and IL-8 were determined via ELISA after **(A, B)** a 4 h and **(C, D)** 48 h incubation following scratching of the cell monolayer and the application of serum free (SF) conditions with or without the supplementation of L4CM. Relative gene expression of IL-6 **(E)** and IL-8 **(F)** in scratched SF HaCaT cells incubated for 4 h with or without L4CM. Data are expressed as fold change compared to SF cells (set to 1). Values of *p* were determined using an unpaired parametric Student’s t-test where **p<0.01 or ****p<0.0001. IL-6, Interleukin 6; IL-8, Interleukin 8.

Protein levels of the secreted inflammatory cytokines IL-6 and IL-8 were assessed 4 h and 48 h after scratching of HaCaT monolayers. Higher concentrations in the L4CM supplemented cells compared with the SF control were observed after 4 h for IL-6 (141 pg/mL vs 25 pg/mL*, p*<0.0001, **Figure 4A**) and for IL-8 (193 pg/mL vs 43 pg/mL, *p*<0.0001, **Figure 4B**). Concentrations of IL-6 and IL-8 were similar in L4CM and SF groups 48 h after scratching (825 pg/mL vs 840 pg/mL, *p*=0.9384 for IL-6 (**Figure 4C**) and 1571 pg/mL vs 2185 pg/mL, *p*=0.1243 for IL-8 (**Figure 4D**)).

Gene expression analysis of IL-6 (**Figure 4E**) and IL-8 (**Figure 4F**) in scratched HaCaTs showed that exposure to L4CM had no significant impact on expression levels at 4 h, but did reduce expression levels at 48 h of IL-6 (0.85-fold change, *p*=0.0593) and IL-8 (0.73-fold change, *p=*0.0062) in comparison with SF cells.

## 4 Discussion

In HaCaT keratinocytes challenged with serum deprivation, metabolites of the Lab4 probiotic consortium (L4CM) were able to prevent loss of metabolic activity, barrier integrity and enhance rates of re-epithelialization while also modulating the acute inflammatory response to damage. These data highlight the potential of Lab4 to support keratinocyte function and augments the growing body of evidence suggesting that supplementation with probiotics can impact upon skin homeostasis and function via the gut-skin axis (Gao et al., 2023).

The removal of serum from HaCaT cells resulted in clear reductions in metabolic activity and apoptosis in alignment with studies performed elsewhere (Lu et al., 2008; Alexaki et al., 2009). In the presence of L4CM, metabolic activity of the serum deprived cells was significantly increased indicating improvement in cellular viability. The improved metabolic activity changes were associated with reductions in apoptotic and necrotic activities. There were also reductions in the gene expression levels of pro-apoptotic Caspase-3 and the BAX/Bcl2 ratio, which in tandem support the anti-apoptotic activity observed with the probiotic. It has been found that GAPDH derived from *Lactobacillus gasseri* can reduce keratinocyte apoptosis via interference of the Caspase-3 cascade and this process may contribute to the mechanism of action for the results obtained with L4CM (Chen et al., 2024).

A robust skin barrier is required to protect the host from environmental insults and to minimize water loss from the body. We found that L4CM supported the maintenance of barrier integrity in serum deprived HaCaT cells (measured via TEER) and increased gene expression levels of the barrier related genes, Filaggrin and Hyaluronan-2 (HAS2). Filaggrin contributes to the formation and maintenance of epidermal barrier integrity (Furue, 2020) and HAS2 contributes to the natural turgidity of the skin barrier and low expression of these genes, as observed in aging skin, is associated with compromised barrier function (Rinnerthaler et al., 2013) and decreased elasticity (Terazawa et al., 2015) respectively. Increased expression levels of Filaggrin and HAS2 have also been observed in keratinocytes with other strains of *Lactobacilli* and *Bifidobacteria* (Kim et al., 2022; Fusco et al., 2023; Zhu et al., 2024).

In order to maintain a robust skin barrier, the epidermis must be able to regenerate and repair effectively after injury - another capability reduced by serum deprivation. It has already been shown that strains of *Lactobacilli* and *Bifidobacteria* can improve re-epithelialization rates in wounded HaCaT monolayers (Lombardi et al., 2019; Brandi et al., 2020) and that probiotic supplementation can enhance skin healing in rats (Tagliari et al., 2022). In this study, the rate of re-epithelialization in serum deprived HaCaT cells was significantly increased in the presence of L4CM and was associated with elevated expression levels of the MMP-1 and HAS-3 genes; MMP-1 encodes for a matrix metalloproteinase enzyme that is vital for matrix remodelling and cleavage of native fibrillar collagens during normal wound resolution (Yeo et al., 2020; Bucekova et al., 2017) and HAS-3 is recognized for its role in the early inflammatory response to injury (Stern et al., 2006).

The wound healing process in skin is orchestrated, at least in part, by inflammatory mediators released by keratinocytes; IL-6 and IL-8 are key pro-inflammatory cytokines produced during the early stages of wound healing and facilitate keratinocyte migration, angiogenesis and recruitment of immune cells such as neutrophils to the wound site (Johnson et al., 2020; Jiang et al., 2012). To explore the impact of L4CM on the inflammatory response to the induced injury the levels of IL-6 and IL-8 secreted by serum deprived HaCaT cells after wounding were measured. Significantly higher levels of IL-6 and IL-8 protein were secreted by cells exposed to L4CM in comparison with the serum free cells within 4 h of wounding suggesting enhancement of the acute inflammatory response. L4CM has been shown to improve the acute immune response of tissue macrophages to both viral and bacterial challenges (Davies et al., 2018). Metabolites generated by *Lactiplantibacillus plantarum* have been shown to improve wound healing *in vitro* and *in vivo* with the latter associated with the upregulation of IL-6 during the early phase of wound healing (Dubey et al., 2021). By 48 h post-wounding the levels of IL-6 and IL-8 protein concentration were found to be similar in both the L4CM and serum free control groups and there were indications of lower expression levels of the IL-6 and IL-8 genes by the L4CM treated cells. Taken together, these data suggest that Lab4 mediated a stronger acute inflammatory response to wounding followed by quicker resolution of wound associated inflammation.

Our study has a number of potential limitations: Firstly, the inflammatory gene markers assessed during the scratch experiments were limited to IL-6 and IL-8 in this study but there are other relevant markers that should be considered for assessment in future work, including other pro-inflammatory cytokines that play a dynamic role in wound healing such as TNF-α (Ritsu et al., 2017), along with growth factors such as TGF-β (Liarte et al., 2020). Secondly, we did not assess the impact of the probiotic in the healthy control HaCaT cells. Finally, the active metabolites generated by Lab4 have not been determined. A recent *in silico* analysis of the Lab4 genome sequences identified the presence of genes involved in the generation of lactic acid, acetic acid and free amino acids (Baker et al., 2021) that have recognized skin benefits (Lew and Liong, 2013., Takaoka et al., 2019).

Future studies are likely to include work with *i*) other epidermal cell lines (Allen-Hoffmann et al., 2000; Baden et al., 1987) and *ii*) 3D skin models/organoids (Lombardi et al., 2024) in order to substantiate our preliminary findings and ultimately *iii*) an intervention study in human subjects. An area of particular interest is the role that probiotics might play in the prevention of skin aging (Waller and Maibach, 2005; Scioli et al., 2014; Bentov and Reed, 2015; Xiao et al., 2023).

In summary, this exploratory study shows that the conditioned media of the Lab4 probiotic consortium helps maintain metabolic activity and barrier integrity, improves the rate of re-epithelialization, and promotes an early immune response to injury in serum deprived HaCaT keratinocytes and therefore suggest that Lab4 may have the ability to support skin function *in vivo.*

## Data Availability

The raw data supporting the conclusions of this article will be made available by the authors, without undue reservation.
