# Supplementary material for: The ability of the Lab4 probiotic consortium to impact upon the functionality of serum deprived human keratinocytes in vitro
Source: Front Microbiomes. 2024 Nov 19;3:1488650. doi: 10.3389/frmbi.2024.1488650 (PMC12993548; doi:10.3389/frmbi.2024.1488650)
Supplement: Supplementary file 1 [file DataSheet1.pdf]

Supplementary Material

1 Supplementary Tables

Table S1. Forward and reverse oligonucleotide primer sequences for RT-qPCR analysis.

Gene	Forward Primer Sequence (5'-3')	Reverse Primer Sequence (5'-3')
Caspase-3	TGCTTCTGAGCCATGGTGAA	CCACGGCAGGCCTGAATAAT
Bax	CCCGAGAGGTCTTTTTCCGAG	CCAGCCCATGATGGTTCTGAT
Bcl2	CATGTGTGTGGAGAGCGTCAA	GCCGGTTCAGGTACTCAGTCA
Filaggrin	TGAAGCCTATGACAC	TCCCCTACGCTTTCT
HAS2	ATTACCCAGTCCTGG	CCTGTGGAAGACTCA
Involucrin	CTGCCTCAGCCTTACTGTGA	GGAGGAGGAACAGTCTTGAGG
Occludin	CGCAGCAGATTGGTT	GATTCGGTTTGAATT
Claudin	CCTGCCCCAGTGGAG	CACCATCAACCCACG
MMP-1	TCTGACGTTGATCCCAGAGAGCAG	CAGGGTGACACCAGTGACTGCAC
MMP-9	GAGACTCTACACCCA	CAAAGTCAAGGGGAA
Ki-67	CCGGATCGTCCCAGTGGA	TTGACACAAAGGAAGCTGGA
IL-6	TCTGCCAGTGCCTCTTTGCT	TTCGGTACATCCTCGACGGC
IL-8	GAAGTTTTTGAAGAGGGCTGAGA	TTTGCTTGAAGTTTCACTGGCA
β-Actin	ACTCTTCCAGCCTTCCTTCC	CGTACAGGTCTTTGCGGATG

2. Supplementary Figures

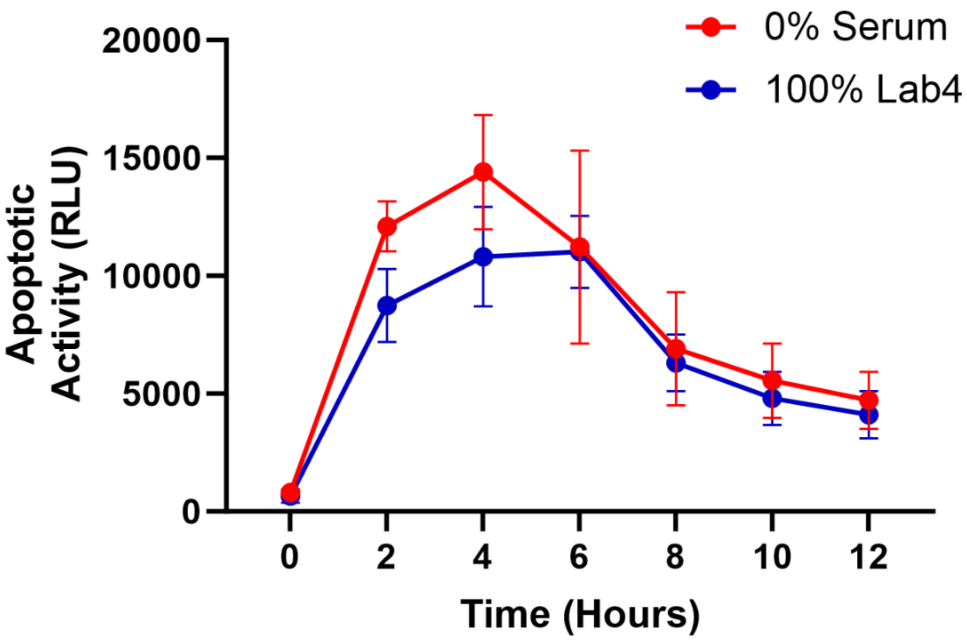

Figure S1. Early apoptotic activity in serum deprived HaCaT cells supplemented with L4CM. Early activity of apoptosis detected in serum deprived HaCaT cells following incubation with L4CM over 12 h. Data are presented as the mean relative luminescence units (RLU) ± standard deviation (SD) from three independent experiments.
